# Alteration in gut microbiota accompanied by increased intestinal permeability and Tfh/Tfr imbalance in patients with active SLE

**DOI:** 10.3389/fcimb.2025.1565416

**Published:** 2025-05-30

**Authors:** Xiaodi Chu, Shuya Li, Yueying Wang, Dazhen Guo, Nana Zhao, Yuanyuan Han, Qian Xing

**Affiliations:** ^1^ Qingdao Municipal Hospital, Qingdao University, Qingdao, Shandong, China; ^2^ School of Clinical Medicine, Shandong Second Medical University, Weifang, Shandong, China; ^3^ Department of Immunology and Rheumatology, Qingdao Municipal Hospital, Qingdao, Shandong, China; ^4^ School of Basic Medical Sciences, Southern Medical University, Guangzhou, Guangdong, China

**Keywords:** gut microbiota, systemic lupus erythematosus, zonulin, Tfh/Tfr, IL-2

## Abstract

**Background:**

Increased intestinal permeability and altered intestinal microbiota may influence cytokine regulatory immunity in systemic lupus erythematosus (SLE). This study aimed to elucidate the relationship between intestinal flora alters and follicular helper T cells (Tfh), regulatory T cells (Treg) cells, and cytokines in SLE.

**Methods:**

In total, 23 patients with active SLE (SLE-A group), 18 patients with stable SLE (SLE-nA group), and 24 healthy controls (HC group) were enrolled. Tfh, follicular T regulatory (Tfr), and Treg cells were measured by flow cytometry, and fecal samples were analyzed using 16S rRNA gene sequencing. The relationship between the gut microbiome and the SLE disease activity index (SLEDAI-2k), zonulin (an indicator of intestinal permeability), IL-2, IL-6, and IL-21 levels was analyzed.

**Results:**

Decreased Treg cells and imbalanced Tfh/Tfr were associated with elevated disease activity in SLE-A group. The increase in zonulin levels in SLE-A group indicated worsened intestinal mucosal barrier damage, potentially linked with the increase in the dominant microflora Escherichia-Shigella. Furthermore, the increase in zonulin was correlated with a severe imbalance in Tfh/Tfr. Moreover, decreased IL-2 levels were associated with a decrease in Ruminococcus and may modulate the reduction in Treg cells during disease progression. Zonulin also exhibited a negative correlation with IL-2.

**Conclusion:**

Zonulin may be involved in the Tfh/Tfr immune imbalance in patients with SLE, and Faecalibacterium and Ruminococcus may contribute to disease development by regulating Treg cells and Tfh/Tfr imbalance. Taken together, these findings may provide new insights into the role of cytokines in the treatment of SLE.

## Introduction

Systemic lupus erythematosus (SLE) exhibits a wide variety of clinical manifestations, and its etiology remains unclear (Li et al., 2019). SLE is a complex disease caused by multiple factors and affects nearly every organ system (Yao et al., 2022). The mechanisms underlying SLE may be influenced by complex genetic, defects in the hormonal, infectious, immune, and environmental factors. Increasing evidence suggests that intestinal barrier caused by an imbalance of intestinal flora may contribute to disease development (Fasano, 2020). Moreover, disease progression has been linked to a “leaky gut” caused by persistent damage to the intestinal barrier. Similarly, it has been demonstrated that patients with SLE exhibit prominent gut microbiota dysbiosis, which primarily manifests as decreased bacterial diversity and altered community composition, especially in patients with active SLE (Li et al., 2019). However, the relationship between changes in intestinal flora and autoimmune activity in SLE remains unclear.

The expansion of detrimental bacterial populations as well as increases in inflammation are what drives”leaky gut” (Di Vincenzo et al., 2024).Consequently, a large quantity of foreign antigens, microorganisms, and their products enter the internal environment of the host through this “leaky gut,” leading to immune activation and inflammation, which promotes the development of SLE (Ma and Morel, 2022). The overexpression and imbalance of T and B cells disrupt adaptive immunity, leading to a disruption in host immune tolerance, which is a core mechanism in SLE progression (Shan et al., 2020). Accordingly, zonulin (the only known physiological regulator of cell-cell tight junctions) has been employed to assess intestinal permeability in various diseases (Gudi et al., 2022). Additionally, its pathogenicity has been implicated or confirmed in chronic inflammatory diseases (Li et al., 2023). Enhanced intestinal permeability has been reported in patients with SLE (Ma and Morel, 2022; Bowes et al., 2024). However, the relationship between intestinal permeability levels, disease activity, and immune cells in SLE remains unexplored.

The gut microbiome has been demonstrated to regulate immune function by influencing the differentiation of lymphocyte subpopulations and their cytokines (Wang et al., 2022). However, the mechanism through which changes in the abundance and diversity of gut microbes cause SLE and how these changes affect disease activity remain unexplored. Therefore, to explore the connection between intestinal flora and disease progression, we aimed to investigate the relationship between intestinal flora and immune cells and cytokines in SLE.

## Materials and methods

### Participants and sample collection

Between September 2022 and July 2024, 41 patients with primary SLE were enrolled from the Department of Immunology and Rheumatology at Qingdao Municipal Hospital, based on the 1997 American College of Rheumatology (ACR) categorization criteria. Additionally, we recruited 24 healthy individuals from the physical examination center as healthy controls (HC). Given that the morbidity of SLE is much higher in females than in males, all patients in this study were female. The participants were from various regions of Shandong Province. The systemic lupus erythematosus disease activity index (SLEDAI-2k) was used to categorize the patients as having either mild or stable SLE (SLE-nA group; SLEDAI<7) or moderate to severe SLE (SLE-A group; SLEDAI≥7). The clinical information of the participants is summarized in **Table 1**. The exclusion criteria were as follows: pregnant or nursing patients, patients with other autoimmune diseases, hypertension, diabetes, severe mental illness, inflammatory bowel disease, other inflammatory diseases, and tumors. Additionally, participants that had received high-dose hormones, oral probiotics, and antibiotics for 8 weeks were excluded. This study was approved by the ethics committee of Qingdao Municipal Hospital, Qingdao University, and each participant voluntarily provided informed written consent to participate in the study. Peripheral blood and fecal samples were collected from each participant.

**Table 1:** Characteristics of the subjects.

	SLE-nA(n=18)	SLE-A(n=23)	HC(n=24)	*p*-value
Age,years	18.28 ± 12.82	22.96 ± 16.46	20.96 ± 11.71	0.565
ESR,mm/h	26.78 ± 15.66	59.04 ± 27.55	6.87 ± 4.85	0.000
CRP,mg/L	9.36 ± 15.42	19.13 ± 37.27	2.61 ± 1.60	0.063
ds-DNA,IU/ml	33.27 ± 22.71	69.12 ± 52.27	1.97 ± 1.19	0.000
24-hour urinary protein quantity,g/24H	0.43 ± 0.20	0.75 ± 0.14	0.11 ± 0.07	0.000
GFR,ml/min·1.73cm^2^	105.67 ± 20.72	91.39 ± 20.63	102.54 ± 9.89	0.024
TG,mmol/L	1.04 ± 0.58	1.30 ± 0.58	0.93 ± 0.44	0.073
Cholesterol	4.69 ± 0.98	4.44 ± 0.91	3.92 ± 0.56	0.021
AST,U/L	22.88 ± 10.71	22.06 ± 11.94	29.09 ± 6.56	0.037
ALT,U/L	24.03 ± 13.96	22.20 ± 25.35	23.10 ± 10.28	0.949
WBC,*10^9^/L	5.17 ± 2.17	3.39 ± 0.87	6.05 ± 1.28	0.000
HB,g/L	124.11 ± 9.63	98.70 ± 25.05	119.96 ± 13.71	0.001
PLT,*10^9^/L	208 ± 92.16	89.26 ± 23.02	228.96 ± 62.95	0.011
C3,g/L	0.75 ± 0.23	0.38 ± 0.18	1.05 ± 0.19	0.000
C4,g/L	0.18 ± 0.99	0.16 ± 0.07	0.28 ± 0.08	0.000
IgG,g/L	14.83 ± 5.75	22.98 ± 7.90	11.35 ± 2.24	0.000
IgM,g/L	0.89 ± 0.51	0.83 ± 0.70	1.22 ± 1.54	0.208

ESR, erythrocyte sedimentation rate; CRP, C-reactive protein; GFR, glomerular filtration rate; TG, triacylglycerol; AST, aspartate transaminase; ALT, alanine aminotransaminase; WBC, leukocyte; RBC, erythrocyte; Hb, hemoglobin; PLT, platelet; C3, complement 3; C4, complement 4; Ig G, immunoglobulin G; Ig M, immunoglobulin M; Ig A, immunoglobulin A.

Whole blood mixed with anticoagulant was stored at 4 °C and centrifuged at 2400 rpm for 10 min. The supernatant was removed and transferred to an Eppendorf tube. Hemolytic serum samples were excluded from our study. Fecal samples were collected in sterile tubes, and all samples were stored in a -80 °C freezer.

### DNA extraction from fecal specimens

Fecal samples were collected using TGuide S96 Magnetic Soil/Stool DNA Kit, according to the supplier instructions. Bacterial DNA was isolated with the TGuide S96 Magnetic Soil/Stool DNA Kit following the manufacturer’s protocol and the V3-V4 hypervariable regions of the 16 S rRNA gene were amplified by paired-end DNA sequencing. What’s more, the primers used for the PCR reactions were 388F (5′-ACTCCTACGGGAGGCAGCA-3′) and 806R (5′-GGACTACHVGGGTWTCTAAT-3′).

### PCR amplification and sequencing

PCR amplification was performed in a mixture containing 50 ng+-20% genomic DNA, 0.3 μL VnF (10 mM), 0.3 μL VnR (10 μM), 5 μL KOD FX Neo Buffer, 2 μL dNTP (2 mM), 0.2 μL KOD FX Neo, and H2O.The reactions were hot-started at 95°C for 5 min, and 95°C for 30s, 50°C for 30s and 72°C for 60s, followed by 10 cycles at 98°Cfor 10s, 65°C for 30s, 72°C for 5min and72°C for 5 min. The products were purified and recovered by the OMEGA DNA Gel Extraction Kit and the Monarch DNA Gel Recovery Kit, sequenced by Illumina Novaseq 6000. Sequence reads were filtered to remove low-quality reads by Trimmomatic (version 0.33). The tag with 97% sequence identity were grouped as operational taxonomical units (OTUs). The taxonomic information of the feature sequences was obtained by comparing against the SILVA database (Release 138.1, http://www.arb-silva.de), using a simple Bayesian classifier (Yao et al., 2022). Alpha and Beta diversity indices were analyzed by QIIME2 and results are displayed using R software. Linear discriminant analysis (LDA) effect size (LEfSe) was used to determine the characteristics of each category.

### Measure of Tfh, Treg, and Tfr cells by flow cytometry

A 100 μL sample of whole blood mixed with anticoagulant was added to a test tube, and 5 μL of each of the following target antibodies was added: CD4 FITC (Immunotech S.A.S.), CD185 (CXCR5) PE (Immunotech S.A.S.), CD278 (ICOS) PE-Cy ANINE5.5 (Immunotech S.A.S.), CD25 PC7 (Immunotech S.A.S.), and CD127 APC (Immunotech S.A.S.). After mixing, the mixture was incubated at 4°C in the dark for 45–60 min. Subsequently, 2 mL of red blood cell lysate was added to the mixture, mixed thoroughly, incubated in the dark at 20–25 °C for 10 min, centrifuged at 1000 rpm for 5 min, and the supernatant was discarded. Following this, 3 mL of PBS (containing 0.1% NaN3) buffer was added, the cells were washed, centrifuged at 1000 rpm for 5 min, and the supernatant was discarded. Finally, 300 μL of fixative was added, and the suspended cells were analyzed using Dx FLEX flow cytometry (Beckman Coulter Ltd., USA) with Cyt Expert software.

### Determination of zonulin and cytokine levels by ELISA

As mentioned in the previous section, serum samples were collected from participants in the SLE-nA, SLE-A, and HC groups. ELISA kits were used to measure zonulin, IL-2, IL-6, and IL-21 levels, according to the manufacturer’s instructions.

### Statistical analysis

Statistical analysis was performed using SPSS version 23.0. Data with a normal distribution were presented using the mean ± standard deviation. Continuous variables were analyzed using variance analysis. Owing to the small sample size, differences in sequencing data were assessed using R language, followed by alpha and beta diversity analysis. The correlation and differences between the data were evaluated using hierarchical clustering, principal coordinate analysis (PCoA), LEfSe analysis, and Spearman correlation analysis.

## Results

### Reduced gut microflora diversity in SLE-A group

**Figure 1:**
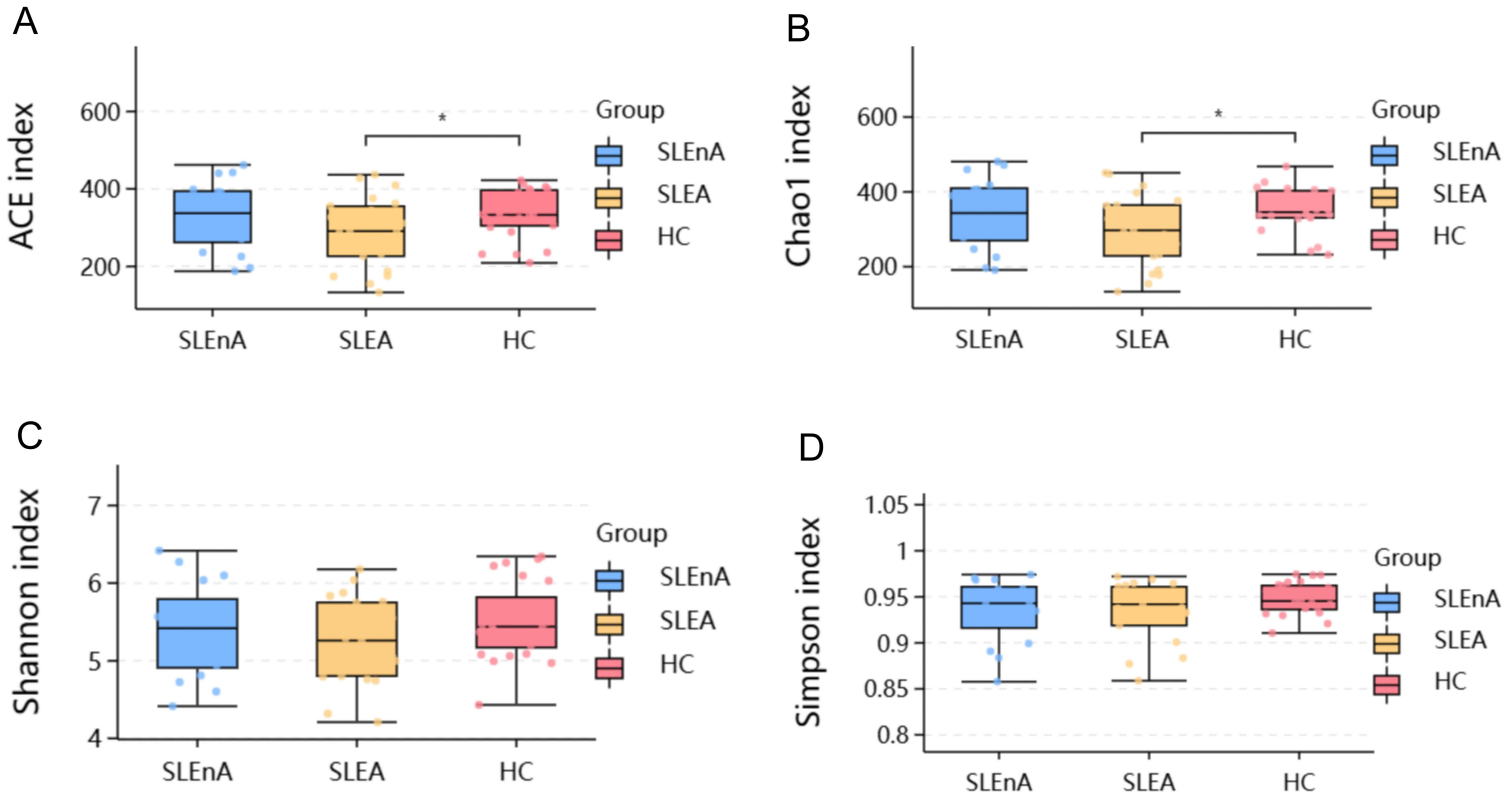
The alpha diversity index of the three groups. **(A)** Ace indexes of the different groups. **(B)** Chao 1indexes of the different groups. **(C)** Shannon indexes of the different groups. **(D)** Simpson indexes of the different groups,*p< 0.05.

Overall, 3570 OTUs in the SLE-A group, 3429 OTUs in the SLE-nA group, and 3805 OTUs in the HC group were identified by analyzing fecal samples from the three groups. The alpha diversity indices were as follows in **Figure 1**: ACE (t=0.357, *p=*0.727), Chao1 (t=0.437, *p=*0.665), Shannon (t=0.641, *p=*0.525), and Simpson (t=1.315, *p=*0.196). Additionally, there were no significant differences in the indices in the SLE-nA with HC and SLE-A groups. However, the ACE (t=2.236, *p=*0.03) and Chao1 (t=2.339, *p=*0.024) indices indicated that the fecal microbial abundance of patients in the SLE-A group was substantially lower than that in the HC group. These findings provide a basis for further analysis of the gut microbiota species.

### Altered microbiota composition among groups

**Figure 2:**
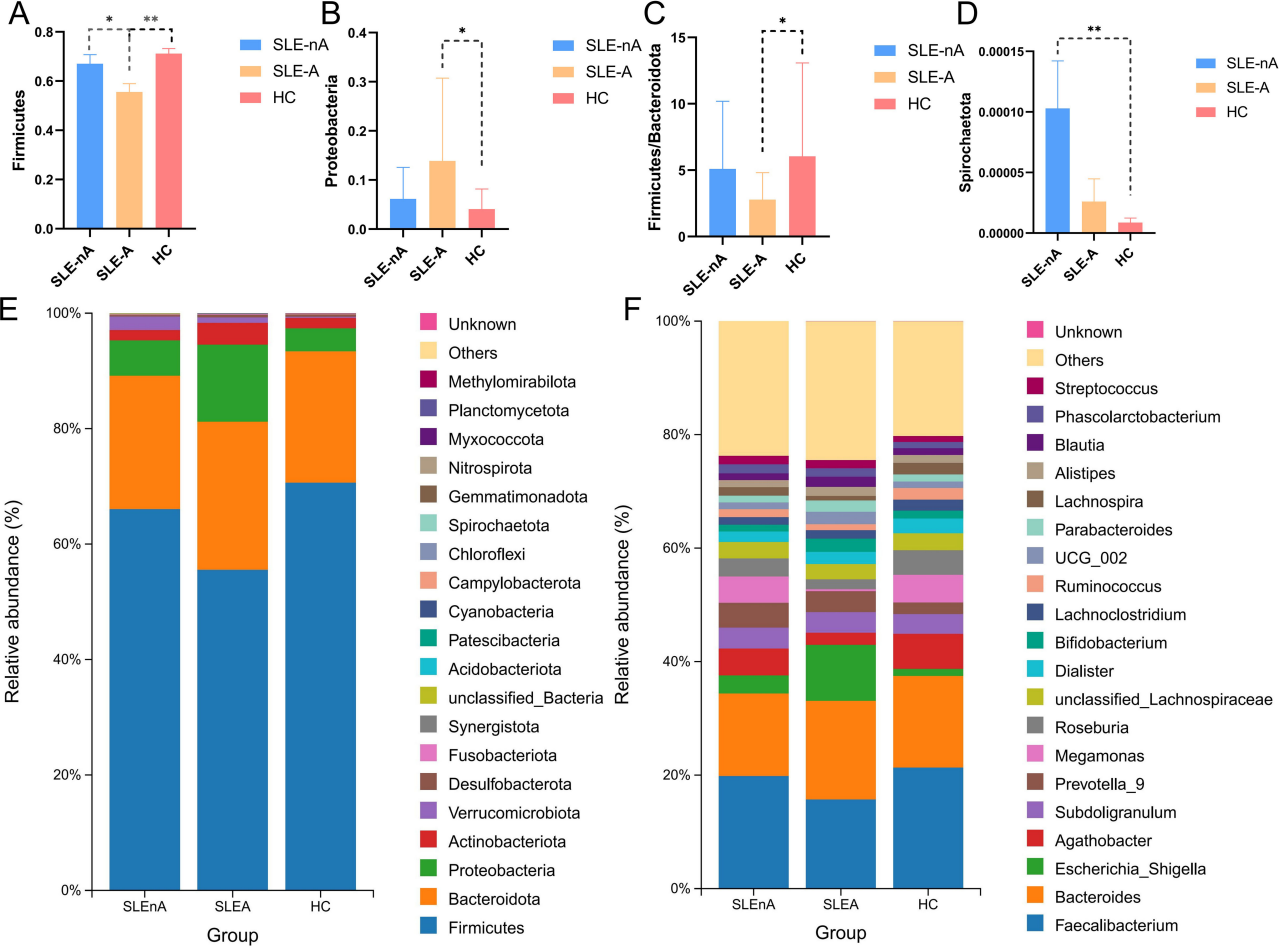
Microbial structures of the three groups. **(A)** Comparison of the three groups of Firmicutes (renamed Bacillota now),*p<0.05**p<0.01. **(B)** Comparison of the three groups of Pseudomonodota, *p<0.05. **(C)** Comparison of the three groups of Firmicutes/Bacteroidota phylum ratios, *p<0.05. **(D)** Comparison of the three groups of Spirochaetota, **p<0.01. **(E)** Relative abundance of gut microbes at phylum levels in the three groups. **(F)** Relative abundance of gut microbes at the genus level.

At the phylum level, the dominant bacteria in the SLE-A, SLE-nA, and HC groups were Bacillota(known as Firmicutes previously), Bacteroidota, and Pseudomonodota(known as Proteobacteria previously). However, the abundance of Bacillota in the SLE-A group was significantly lower than that in the HC group, while that of Pseudomonodota was significantly higher (**Figure 2E**). The microbial composition at the phylum level was compared between the three groups using the Spearman test, which revealed that, compared with the SLE-A group, Bacillota (x^2^ = 11.73, *p*<0.01; **Figure 2A**), Pseudomonodota (x^2^ = 6.729, *p*<0.05; **Figure 2B**), and Firmicutes/Bacteroidota (F/B) (*p*<0.05; **Figure 2C**) were decreased. Additionally, compared with the HC group, the abundance of Spirillobacteria (x^2^ = 7.038, *p*<0.05; **Figure 2D**) was higher in the other groups. Additionally, the abundance of Bacillota was higher in the SLE-nA group than the SLE-A group (*p*<0.05).

At the genus level, the dominant genera in the SLE-A group were Faecalibacterium, Bacteroides, and Escherichia-Shigella, while the dominant genera in the SLE-nA and HC groups were Faecalibacterium, Bacteroides, and Agathobacter (**Figure 2F**). Compared with the HC group, the abundance of Faecalibacterium (*p=*0.058), Agathobacter (*p*<0.01), Ruminococcus (*p*<0.05), and Megamonas (*p=*0.121) were significantly lower in the SLE-A group, while Escherichia-Shigella (*p*<0.05) was significantly higher.

**Figure 3:**
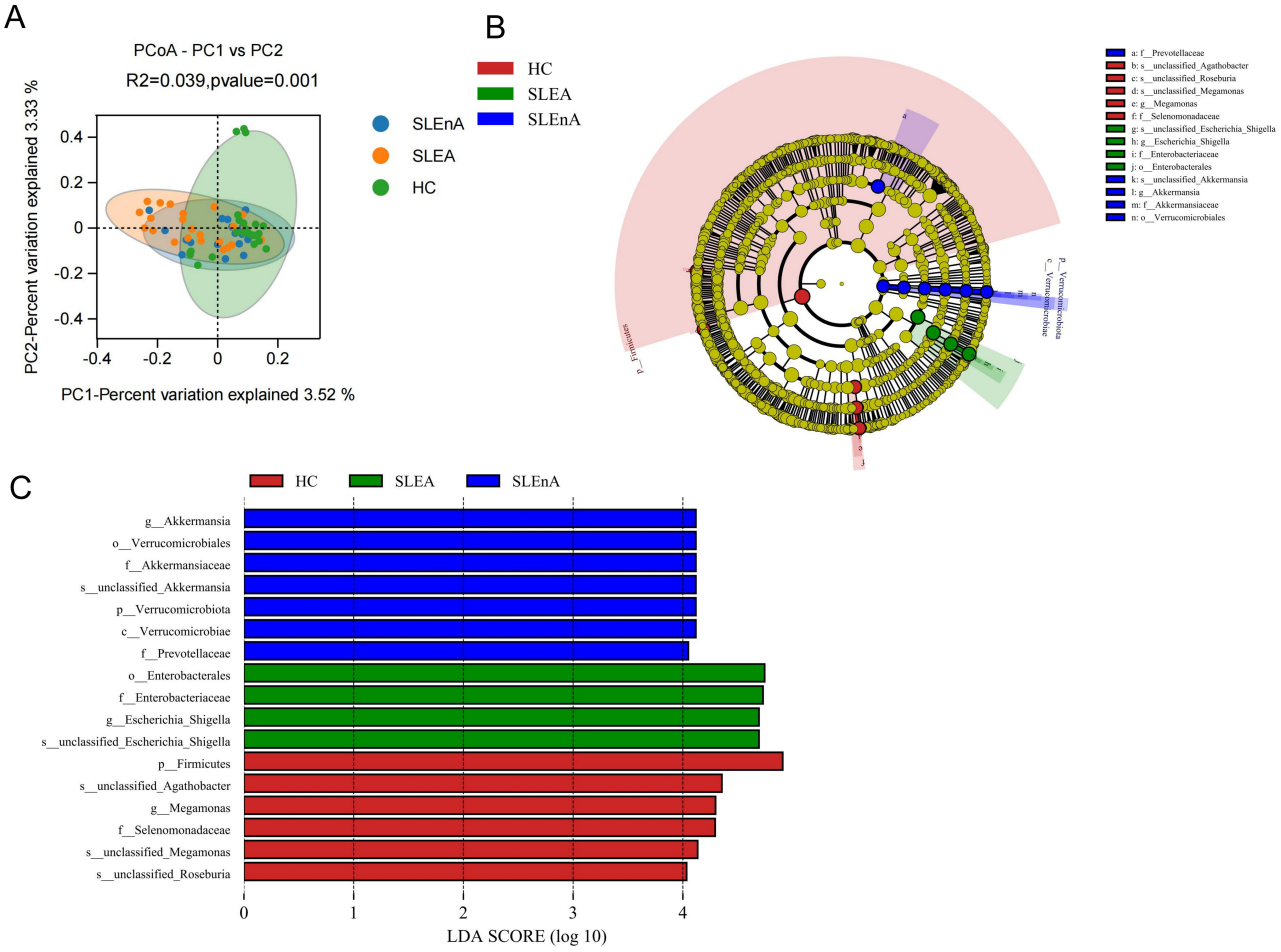
**(A)** At the OTU level, the principal coordinates analysis (PCoA) revealed fecal microbiota compositions in the three groups based on the Bray-Curtis distance. r^2^ = 0.039, *p=*0.001. **(B)** Cladogram from phylum to genus levels of the LEfSe analysis. **(C)** Histogram of the LDA scores (LDA >4) for different representative genera in HC, the SLE-d group, and the SLE-nd group.

At the OTU level, statistically significant differences in beta diversity, based on the Bray-Curtis distance, between the three groups were observed in the PCoA analysis (*p=*0.001; **Figure 3A**).

Cladoplots showing the unique fecal microbial taxa from the phylum to the genus level from all three groups were generated using the LEfSe method. In the SLE-nA group, Akkermansia (LDA 4.12) and Prevotellacea (LDA 4.05) were the predominant bacterial groups. Conversely, in the SLE-A group, Escherichia-Shigella (LDA 4.69) and Enterobacterales (LDA 4.74) were the predominant bacterial groups. Meanwhile, in the HC group, Megamonas (LDA 4.30) and Selenomonadaceae (LDA 4.29) were the predominant bacteria (**Figures 3B, C**). Moreover, LEfSe analysis revealed potential biomarkers with statistically significant differences.

### The association of peripheral blood zonulin with SLEDAI-2k and IL-2 and IL-21 levels in the SLE-A group

**Figure 4:**
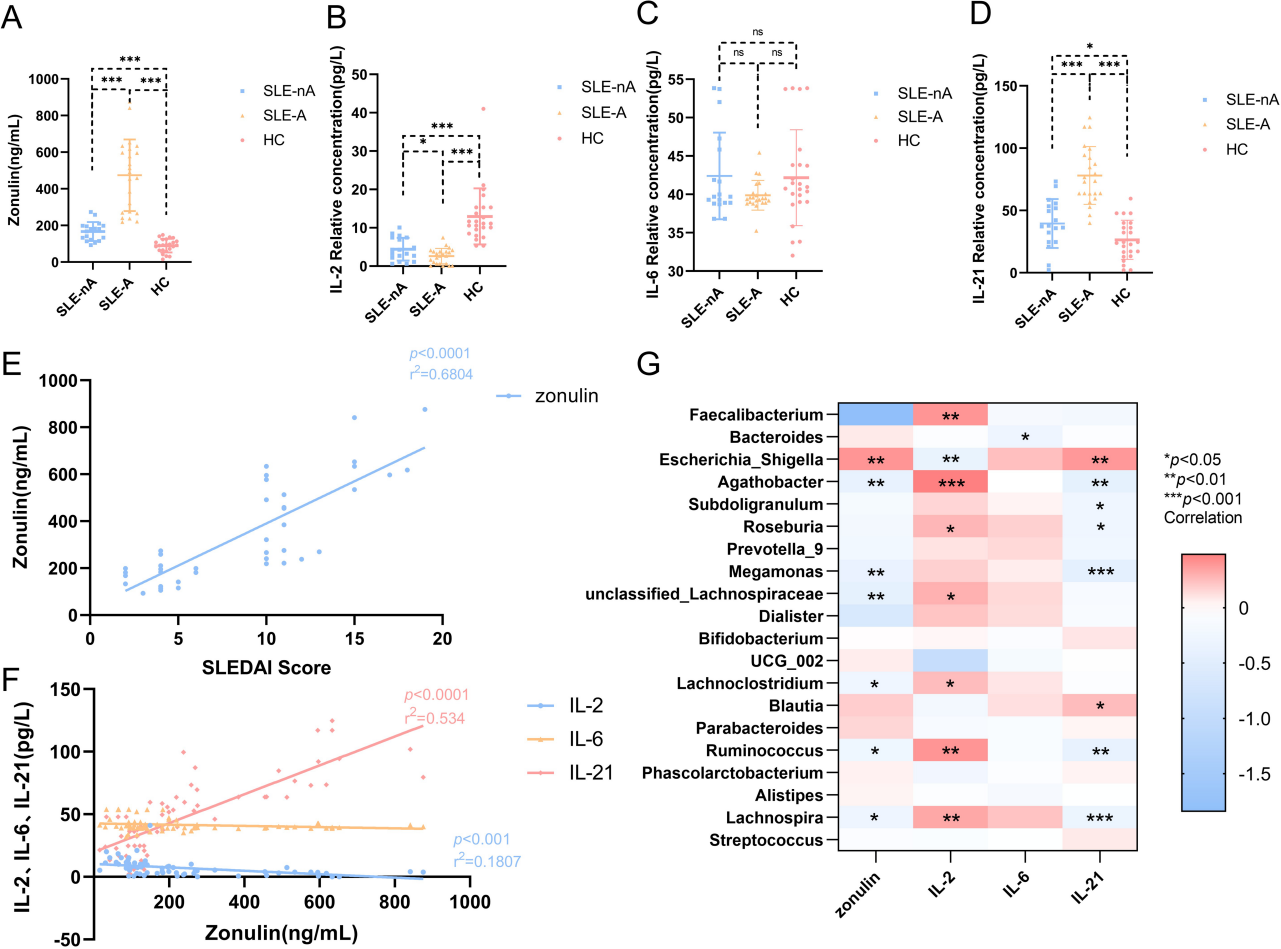
**(A)** Serum Zonulin levels in different groups,***p<0.001 **(B)** Serum IL-2 levels in different groups,*p<0.05***p<0.001 **(C)** Serum IL-6 levels in different groups **(D)** Serum IL-21 levels in different groups, *p<0.05***p<0.001 **(E)** Scatter plot depicting the correlation between plasma zonulin levels and SLEDAI scores. The x-axis corresponds to SLEDAI scores, while the y-axis refers to the zonulin concentrations. The r^2^ value indicates the directionality of the relationship. A *p*<0.05 was considered statistically significant. **(F)** Scatter plot depicting the correlation between plasma serum IL-2, IL-6, IL-21 levels and the zonulin concentrations. The x-axis corresponds to the zonulin concentrations, while the y-axis refers to serum IL-2, IL-6, IL-21 levels. The r^2^ value indicates the directionality of the relationship. A *p*<0.05 was considered statistically significant. **(G)** Relationship between gut microbiota and cytokines. Heat map of the correlation between different gut microbial species and cytokines. Colors indicate the Spearman rank correlation, with lighter and darker colors corresponding to weaker and stronger correlations, respectively. *p< 0.05**p< 0.01***p<0.001.

To evaluate the intestinal mucosal barrier function and inflammatory status of patients in the SLE-nA, SLE-A, and HC groups, we measured zonulin, IL-2, IL-6, and IL-21 levels. Zonulin was significantly higher in the SLE-A and SLE-nA groups than in the HC group (474.40 ± 196.56, 167.06 ± 51.39 vs. 89.95 ± 36.73, *p*<0.001). Moreover, zonulin was significantly higher in the SLE-A group than in the SLE-nA group (*p*<0.001; **Figure 4A**). Conversely, IL-2 levels in the SLE-A group were significantly lower than those in the SLE-nA (2.70 ± 2.03 vs. 4.39 ± 2.97, *p*<0.05) and HC groups (2.70 ± 2.03 vs. 13.27 ± 7.29, *p*<0.001). Furthermore, IL-2 levels in the SLE-nA group were significantly lower than those in the HC group (*p*<0.001; **Figure 4B**). Regarding IL-21, the SLE-A group exhibited significantly higher IL-21 levels than the SLE-nA (78.08 ± 23.18 vs. 39.56 ± 19.57, *p*<0.001) and HC groups (78.08 ± 23.18 vs. 26.45 ± 15.70, *p*<0.001). Furthermore, IL-21 levels in the SLE-nA group were higher than those in the HC group (39.56 ± 19.57 vs. 26.45 ± 15.70, *p*<0.05; **Figure 4D**). However, there was no significant difference in IL-6 levels between the three groups (**Figure 4C**).

Simultaneously, we explored the correlation between intestinal mucosal barrier function and serum inflammatory factors. We revealed that zonulin was positively correlated with the SLEDAI score (r^2^ = 0.6229, *p*<0.001; **Figure 4E**). However, zonulin was negatively correlated with the serum inflammatory factor IL-2 (r^2^ = 0.7695, *p*<0.001; **Figure 4F**) and positively correlated with IL-21 (r^2^ = 0.534, *p*<0.001; **Figure 4F**).

### Correlations between gut microbes and serum zonulin, IL-2, IL-6, and IL-21 levels

Furthermore, we investigated the relationship between zonulin, serum autoinflammatory factors, and the relative abundance of various bacterial genera. Zonulin was positively associated with Escherichia-Shigella (r=0.398, *p*<0.01) and negatively correlated with Agathobacter (r=-0.387, *p*<0.01), Megamonas (r=-0.381, *p*<0.01), unclassified-Lachnospiraceae (r=-0.420, *p*<0.01), Lachnoclostridium (r=-0.273, *p*<0.05), Ruminococcus (r=-0.28, *p*<0.05), and Lachnospira (r=-2.93, *p*<0.05). Furthermore, IL-2 was positively correlated with Faecalibacterium (r=-0.401, *p*<0.01), Agathobacter (r=0.481, *p*<0.001), Roseburia (r=0.274, *p*<0.05), unclassified-Lachnospiraceae (r=0.293, *p*<0.05), Lachnoclostridium (r=0.251, *p*<0.05), Ruminococcus (r=0.401, *p*<0.01), and Lachnospira (r=0.328, *p*<0.01) and negatively correlated with Escherichia-Shigella (r=-0.368, *p=*0.001). Meanwhile, IL-21 was positively correlated with Escherichia-Shigella (r=0.384, *p*<0.01) and Blautia (r=0.25, *p*<0.05) and negatively correlated with Agathobacter (r=-0.416, *p*<0.01), Subdoligranulum (r=-0.259, *p*<0.05), Roseburia (r=-0.267, *p*<0.05), Megamonas (r=-0.438, *p*<0.001), Ruminococcus (r=-0.401, *p*<0.01), and Lachnospira (r=-0.325, *p*<0.001). IL-6 was negatively associated with Bacteroides (r=-0.289, *p*<0.05; **Figure 4G**).

### Analysis of Tfh, Treg, and Tfr cells by flow cytometry

**Figure 5:**
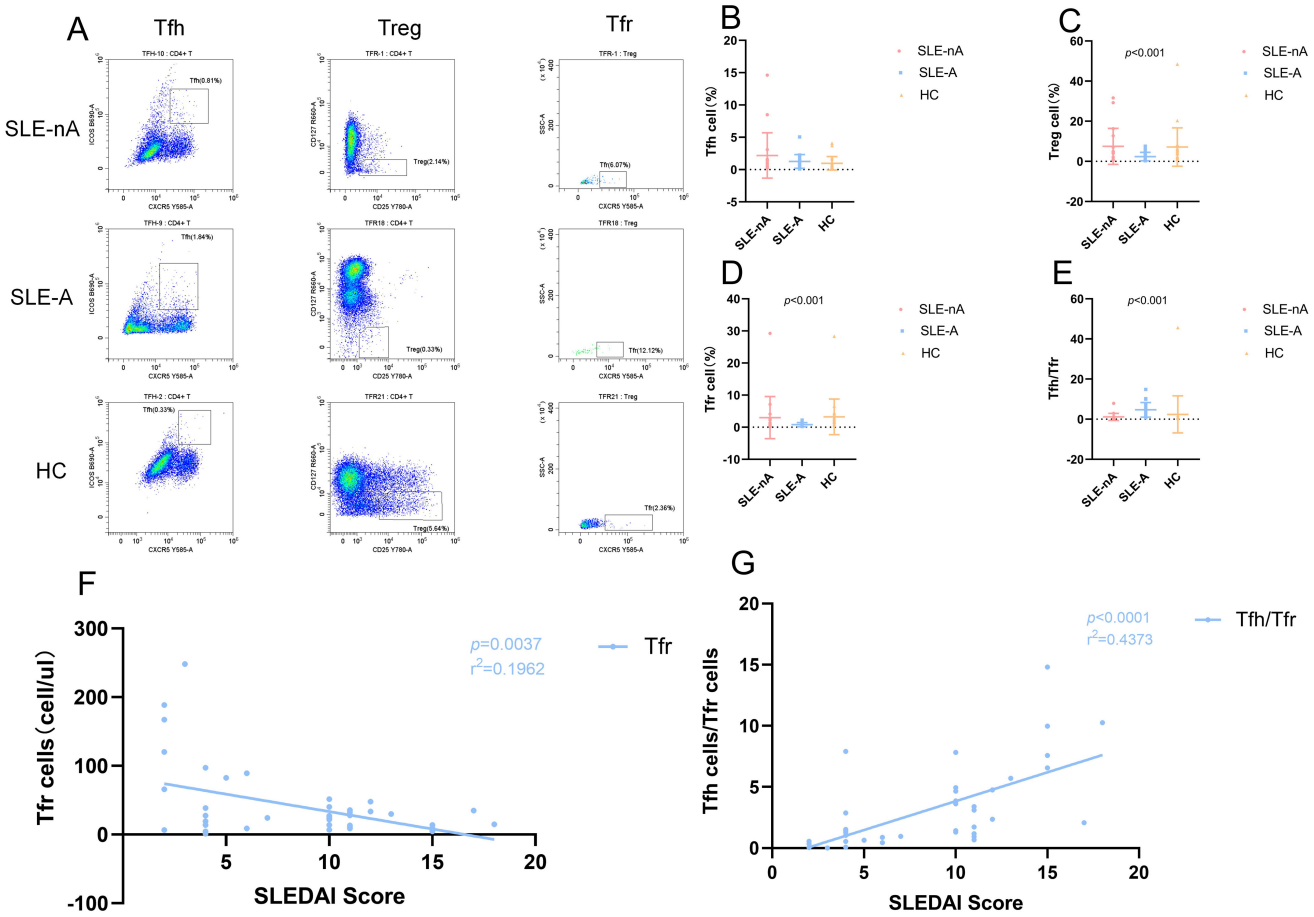
**(A)** Representative flow cytometry dot plots illustrate the expression of Tfh (CD4^+^CXCR5^+^ICOS^+^), Treg (CD4^+^CD25^hi^CD127^low^) and Tfr (CD4^+^CD25^hi^CD127^low^CXCR5^+^) cells in SLE-nA, SLE-A and HC groups. The rows means SLE-nA, SLE-A, HC group, and the columns means Tfh, Treg and Tfr cells. **(B)** Percentage of circulating Tfh (CD4^+^CXCR5^+^ICOS^+^) cells in SLE-A, SLE-nA groups and healthy controls. **(C)** Percentage of circulating Treg (CD4^+^CD25^hi^CD127^low^) cells in SLE-A, SLE-nA groups and healthy controls. **(D)** Percentage of circulating Tfr (CD4^+^CD25^hi^CD127^low^CXCR5^+^) cells in SLE-A, SLE-nA groups and healthy controls. **(E)** The Tfh/Tfr ratio in SLE-A, SLE-nA and HC. **(F)** The correlation of SLEDAI with the Tfh and Tfr cells in SLE patients(n=41). The r^2^ value indicates the directionality of the relationship. A *p*<0.05 was considered statistically significant. **(G)**The correlation between SLEDAI and Tfh/Tfr ratio(n=41). The r^2^ value indicates the directionality of the relationship. A *p*<0.05 was considered statistically significant.

The precursor cells of Tfh and Tfr cells cannot be detected in the bloodstream owing to the lack of specific cell surface markers. However, T cells differentiate into Tfh and Tfr cells with memory phenotypes upon migration to the germinal center, making them easier to detect in circulation. First, lymphocytes are identified from whole blood cells and CD4^+^T cells are selected from them. According to our gating strategy, Tfh cells are CXCR5^+^ICOS^+^ T cells in CD4^+^T cells. Treg cells are CD25^hi^CD127^low^T cells in CD4^+^T cells. Since Tfr cells marked as CD4^+^CXCR5^+^CD25^hi^CD127^low^, CXCR5^+^ cells consider as Tfh cells in Treg cells. Representative flow cytometric graphs of Tfh (CD4^+^CXCR5^+^ICOS^+^), Treg (CD4^+^CD25^hi^CD127^low^) and Tfr (CD4^+^CXCR5^+^CD25^hi^CD127^low^) cells in SLE-nA, SLE-A and HC groups are shown in **Figure 5A**.

The frequency of Tfh, Treg, and Tfr cells and the Tfh/Tfr ratio were analyzed using the rank sum test. We found the absolute value of Tfh cells were more in the SLE-A group than in the HC group (**Figure 5B**). Although the absolute value of the SLE-A group exhibited a substantially higher number of Tfh cells, the overall proportion of Tfh cells was not significantly different across the three groups. In contrast, the Treg (*p*<0.001; **Figure 5C**) and Tfr (*p*<0.001; **Figure 5D**) cells and the Tfh/Tfr ratio (*p*<0.001; **Figure 5E**) differed significantly. Specifically, the frequencies of Treg and Tfr cells in the SLE-A group were lower than those in the SLE-nA group, while those in the SLE-nA group were lower than those in the HC group. We further investigated the correlation between the SLEDAI score and the absolute values of Tfh, Treg, and Tfr cells, as well as the Tfh/Tfr ratio. We revealed that the SLEDAI-2k score had a negative correlation with Tfr cells (r^2^ = 0.1692, *p=*0.0037) (**Figure 5F**) and a positive correlation with the Tfh/Tfr ratio (r^2^ = 0.4373, *p*<0.001) (**Figure 5G**), which is consistent with previous studies. However, there was no significant correlation between the SLEDAI-2k score and Tfh and Treg cells.

### Correlation between Tfh, Treg, and Tfr cells and IL-2, IL-6, and IL-21

**Figure 6:**
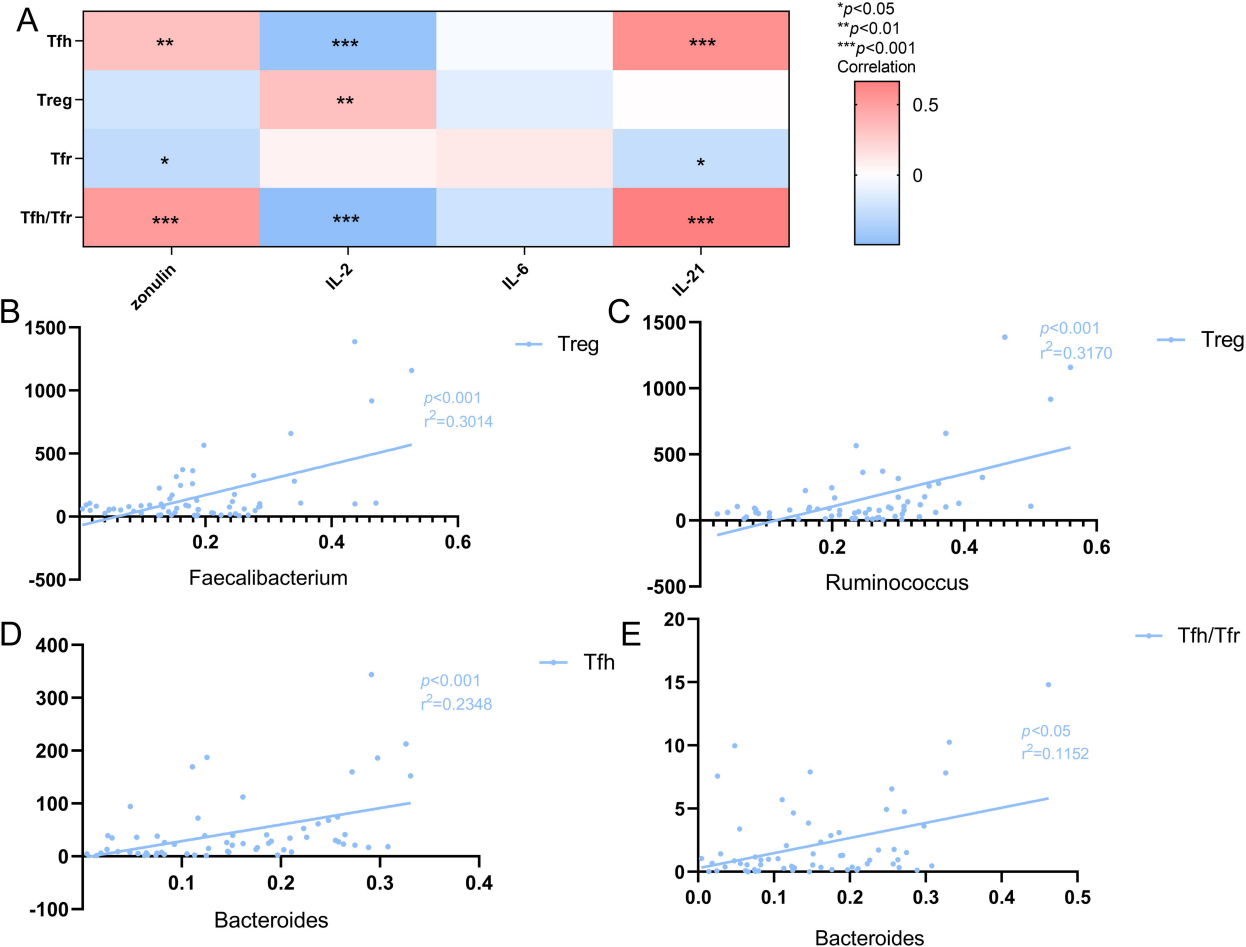
**(A)** Heat map of the correlation between Tfh cells, Treg cells, Tfr cells and Tfh/Tfr ratio and cytokines. Colors indicate the Spearman rank correlation, with lighter and darker colors corresponding to weaker and stronger correlations, respectively.*p<0.05**p<0.01 ***p<0.001. **(B)** The correlation of Treg cells with the Faecalibacterium in SLE patients(n=41).The r^2^ value indicates the directionality of the relationship. A *p*<0.05 was considered statistically significant. **(C)** The correlation of Treg cells with the Ruminococcus in SLE patients(n=41).The r^2^ value indicates the directionality of the relationship. A *p*<0.05 was considered statistically significant. **(D)** The correlation of the Bacteroiades with Tfh cells in SLE patients(n=41).The r^2^ value indicates the directionality of the relationship. A *p*<0.05 was considered statistically significant. **(E)** The correlation of the Bacteroiades with Tfh/Tfr ratio in SLE patients(n=41).The r^2^ value indicates the directionality of the relationship. A *p*<0.05 was considered statistically significant.

The results showed that the number of Tfh cells was positively correlated with peripheral blood zonulin (r=0.328, *p <*0.01) and IL-21 (r=0.57, *p*<0.001) protein levels and negatively correlated with IL-2 levels (r=-0.462, *p*<0.001). Conversely, the number of Treg cells was positively correlated with IL-2 levels (r=0.324, *p*<0.01). The number of Tfr cells was negatively associated with zonulin (r=-0.279, *p*<0.05) and IL-21 levels (r=-0.258, *p*<0.05). Furthermore, there was no significant correlation with IL-2, IL-21, and zonulin. However, the Tfh/Tfr ratio exhibited a positive correlation with zonulin (r=0.520, *p*<0.001), IL-2 (r=-0.493, *p*<0.001), and IL-21 levels (r=0.663, *p*<0.001). Moreover, there was no obvious correlation between IL-6 and these three immune cells, as well as the Tfh/Tfr ratio (**Figure 6A**).

Further analysis involving the dominant and characteristic flora revealed a positive correlation between Faecalibacterium and Treg cells (r^2^ = 0.3014, *p*<0.001; **Figure 6B**), and between Bacteroiades and Tfh cells (r^2^ = 0.2348, *p*<0.001; **Figure 6D**), as well as the Tfh/Tfr ratio (r^2^ = 0.1125, *p*<0.05; **Figure 6E**). Additionally, Ruminococcus was positively correlated with the number of Treg cells (r^2^ = 0.3170, *p*<0.001; **Figure 6C**).

## Discussion

Our findings reveal that the gut microbiota composition, as well as the alpha and beta diversity, are altered in patients with SLE, which is similar to previous studies (Li et al., 2019). Reduced alpha diversity (as indicated by Chao 1 and ACE indices) in patients with SLE, particularly those with medium and severe SLE, indicates lower richness and imbalance in their gut microbiota. Similarly, the beta diversity (PCoA) results also revealed disruptions in the community (Kalayci and Ozen, 2023), with patients with SLE exhibiting significant differences in gut microbiota composition compared to healthy populations (Widhani et al., 2022). At the phylum level, the microbiota was mainly composed of Bacillota, Bacteroidota, and Pseudomonodota; however, in patients with severe SLE, Bacillota were significantly reduced, while there was a higher abundance of Bacteroidota, Pseudomonodota, and Actinobacteria (Ling et al., 2023). Bacillota can produce butyrate, which alters T cell function by inhibiting histone deacetylases and suppressing Treg cell differentiation, which regulates inflammation, thereby promoting disease progression (Abdelhamid et al., 2020). The significant decrease in the phylum of Bacillota in the moderate to severe SLE group provides clinical support for the possibility that the Bacillota may affect disease activity by this way. Previous studies involving patients with other conditions have indicated that there is some imbalance between Bacillota and Bacteroidota in the gastrointestinal tract (Yao et al., 2023), with a lower Firmicutes/Bacteroidota (F/B) ratio in patients with SLE, especially severe SLE (Fasano, 2020). Although some studies have reported that the F/B ratio in patients with SLE does not differ from that in the gut microbiota of healthy individuals, a large number of research have provided the dysregulation of F/B ratio in the gut microbiota of SLE patients (Yao et al., 2023). SLE is regulated by diet, drug treatment, and environmental microbes (Li et al., 2021). Therefore, the pathogenesis of SLE has been studied using mouse models that simulate the intestinal flora of patients with SLE (Yao et al., 2023). Furthermore, the dynamics of the gut microbiome in SLE have been explored using classical autoimmune SLE models (MRL/lpr or NZB/W F1), which exhibit a phenotype that resembles human SLE (Wang and Deng, 2024). Notable differences in the intestinal flora of NZB/W F1 mice have been observed, with substantial changes in the abundance of several representative bacterial species observed between the premorbid and onset stages of SLE (Wang et al., 2021). Abdelhamid et al. found a positive correlation between the abundance of Bacteroidota and glomerular pathological scores (Abdelhamid et al., 2020). Moreover, the lower F/B ratio observed in 6-week-old MRL/lpr mice indicates that the F/B ratio may play a notable role in promoting early disease onset that cannot be overlooked (Luo et al., 2018). Therefore, further research is still warranted to investigate the influence of the F/B ratio in the pathogenesis of SLE.

We observed that the dominant bacteria in the SLE-A group were Faecalibacterium, Bacteroides, and Escherichia-Shigella, while the dominant bacteria in the SLE-nA and HC groups were Faecalibacterium, Bacteroides, and Agathobacter. Compared to the HC group, Faecalibacterium(belonging to Bacillota) and Ruminococcus were significantly less abundant. Similar studies have demonstrated that, in other immune-related diseases, the abundance of Faecalibacterium may enhance epithelial permeability, leading to microbial products appearing in the lamina propria and subepithelial space (Moosavi et al., 2020). This indicates a potential correlation between the intestinal mucosal barrier function of zonulin and Faecalibacterium, which may align with our findings (Ma and Morel, 2022). A recent study found that a strain of Ruminococcus, which belongs to Bacillota, induced increased intestinal permeability in C57BL-6 mice, elevated serum zonulin levels, and was significantly associated with SLE biomarkers such as dsDNA antibodies. Additionally, an expansion of obligate anaerobic Ruminococcus was observed in patients with active-stage lupus nephritis (Bowes et al., 2024). However, the specific mechanism and role of Bacillota and its derived bacteria, particularly in relation to renal involvement in SLE, warrant further study.

The intestinal epithelial barrier constitutes the primary defense against luminal antigens. Gastrointestinal barrier dysfunction in SLE permits microbial translocation into systemic circulation, triggering sustained immune activation through pathogen-associated molecular patterns (PAMPs) (Wen et al., 2024). SLE patients demonstrate concomitant gut dysbiosis (Toumi et al., 2023), and impaired mucosal integrity. Emerging evidence suggests plasma microbiome-derived components accelerate lupus progression via proinflammatory cytokine induction rather than direct microbial effects (Liu et al., 2022), perhaps induced by Toll-like receptor agonists in the plasma microbiome (Silverman et al., 2022). Furthermore, we report a correlation between intestinal mucosal barrier function, indicated by plasma zonulin levels, and findings observed in a clinical study (with no male patients), which closely aligns with previous studies (Li et al., 2023). Studies have confirmed that in lupus patients, intestinal permeability is positively correlated with disease activity, and the DNA methylation level of 926 CpG sites is significantly related to intestinal permeability. Increased intestinal permeability may be associated with epigenetic changes, which may play a role in the pathogenesis of lupus (Bowes et al., 2024). Zonulin exhibited a negative correlation with IL-2 and a positive correlation with IL-21. IL-2 is involved in the synthesis and expression of its high-affinity receptor as well as the IL-2-sensitive response; therefore, it plays a crucial role in the development, survival, maintenance, and function of Treg cells (Xu et al., 2017). Treg cells maintain intestinal immune tolerance through symbiotic interactions with commensal microbiota and dietary antigens (Ramanan et al., 2023), while simultaneously enforcing peripheral self-tolerance mechanisms (Zhang et al., 2024). Microbiota-dependent RORγ^+^ Treg subsets specifically regulate bacterial tolerance, potentially via MAF-mediated suppression of effector T cell cytokines (Xu et al., 2018).

Recent studies have shown that low-dose IL-2 can alter the balance between autoimmune response and immunological tolerance by promoting Treg cell expansion and inhibiting Tfh cell differentiation. It has been demonstrated that IL-2 could inhibit Th17 and Tfh cell differentiation and promote Treg, Th1, and Th2 cell lineage commitment. Since Th17 and Tfh cells are involved in various autoimmune diseases (Saravia et al., 2019). Mechanistically, IL-2 activates the JAK-STAT5 pathway to upregulate B lymphocyte-induced maturation protein 1 (Blimp1), which antagonizes B-cell lymphoma 6 (Bcl-6)-mediated Tfh cell development (Tokura, 2012). What’s more, the diminished CD25 expression on Tfh precursor cells creates a permissive microenvironment for Bcl-6 overexpression, thereby amplifying Tfh differentiation through Blimp1 suppression (Singh et al., 2014). Clinical evidence demonstrates that low-dose recombinant human IL-2 therapy selectively expands Treg populations while reducing pathogenic Th17 and Tfh cells, correlating with significant attenuation of SLE disease activity (He et al., 2016). These findings provide new insights for improving the immune status of patients with SLE (Zhang et al., 2024).

Tfh cells and their progenitors secrete substantial quantities of IL-21, and it has been demonstrated that IL-21 plays a significant role in the pathogenesis of SLE (Jin et al., 2022). IL-21 exhibits diverse immunoregulatory functions in both B and T cells, including Tfr cells (Huang et al., 2024). Significantly elevated plasma IL-21 levels were observed in patients with SLE compared to individuals in the HC group. Moreover, plasma IL-21 levels exhibited a negative correlation with the proportion of Tfr cells but were positively correlated with both Tfh cell numbers and the Tfh/Tfr ratio. Bacteroides was positively correlated with the absolute Tfh cell count and the Tfh/Tfr ratio, while Ruminococcaceae was correlated with Treg cells, indicating that changes in the intestinal flora are closely linked to a shift in the balance between pro-inflammatory and anti-inflammatory T cells in SLE. This highlights that a dysfunction in intestinal flora plays a crucial role in the immune mechanism underlying the pathogenesis of SLE. Previous studies have also reported that Ruminococcaceae is significantly associated with the Th1/Th2 and Th17/Treg ratios but not the number of Th1, Th2, or Th17 cells, which may be linked to changes in the intestinal microbial population of patients with SLE (Hu et al., 2024). Our results show that Bacteroides is directly correlated with the absolute value of Tfh cells and the Tfh/Tfr ratio but has no correlation with the absolute value of Tfr cells, and Ruminococcus is positively correlated with Treg cells. Therefore, the abundance of Bacteroides and its associated flora may influence the pathogenesis of SLE by affecting Tfh cells and the Tfh/Tfr ratio, and the absolute Treg count in peripheral blood may be modulated by the abundance of specific bacteria such as Ruminococcus, thus contributing to the pathogenesis of SLE.

Although this study further validates the involvement of intestinal commensal flora in the pathogenesis of SLE through changes in the abundance of intestinal flora and lymphocyte subset levels, further in-depth research is needed to explore how flora imbalances and immune cells are involved in the pathogenesis and progression of SLE.

Both mouse models and human studies demonstrate that Tfh cells play an important role in autoantibody responses in SLE. Research has shown that patients with SLE have decreased Tfr frequency and an increased Tfh/Tfr ratio, and Tfr cell frequency is negatively associated with disease activity, dsDNA levels, and the Tfh/Tfr ratio (Zhang et al., 2015). Subsequently, immunological results from a non-controlled single-center study revealed that treatment with low doses of IL-2 led to a decrease in the percentage of Tfh cells among CD4 T cells (the effect on the absolute numbers of these subpopulations was not reported).

Historically, IL-2 has been implicated as a T cell growth factor whose primary function is to promote the proliferation and activation of effector T cells and possibly induce an autoimmune response (Zhou et al., 2023). However, this view has been challenged following studies using mice deficient in IL-2 or IL-2Rα (CD25) genes, suggesting that the primary function of IL-2 *in vivo* may be to maintain autoimmune tolerance rather than immune support and enhancement (Xie et al., 2021). Recent studies indicate that IL-2 is a key cytokine for maintaining differentiation, proliferation, and function in Treg cells (Zhou et al., 2023). Furthermore, clinical trials have demonstrated that low doses of IL-2 are safe and selectively promote thymus-derived Treg cells, thereby demonstrating significant clinical efficacy on active and refractory SLE by enhancing the treatment population (Raeber et al., 2024).The variability in clinical response in different patient subgroups may be attributed to differences in Treg cell deficiency or the role Tregs play in disease development. However, further research is warranted to identify molecules for low-dose IL-2 treatment, biomarkers that predict biological and clinical responses to low-dose IL-2 treatment, and establish appropriate individual criteria to stratify patients.

Our results indicated that IL-2 levels are associated with the absolute Treg and Tfh cell counts and the Tfh/Tfr ratio, and they also have a positive relationship with Faecalibacterium and Ruminococcus. Therefore, we performed linear regression analysis on the absolute values of Treg cells with Faecalibacterium and Ruminococcus, and we revealed there was a positive correlation between them. Therefore, Faecalibacterium and Ruminococcus may serve as potential biomarkers to stratify patients for low-dose IL-2 treatment for patients with SLE. However, further research is warranted to validate this. Our research suggests that the dominant and specific bacterial genera may serve as potential biomarkers to stratify patients for low-dose IL-2 treatment for SLE.

Our study had certain limitations. First, our study was only conducted in Shandong Province in China; multiregional surveys may provide more comprehensive findings in future research. Second, the sample size of our study is small, which may introduce some error; studies with a larger sample size are necessary to obtain more accurate and meaningful results. Thirdly, although strictly dietary habits had been required in patients, the differences in individual dietary habits may affect the accuracy of the study findings. Finally, owing to laboratory conditions and time constraints, this study did not further explore the relationship between the metabolic pathways of intestinal flora, immune cells, and cytokines, and we could not conduct *in vitro* tests, limiting our understanding and analysis of the interaction mechanisms between intestinal flora, immune cells, and cytokines.

## Data Availability

The datasets presented in this study can be found in online repositories. The names of the repository/repositories and accession number(s) can be found below: NCBI SRA, PRJNA1266339.
